# Fecal calprotectin in juvenile idiopathic arthritis patients related to drug use

**DOI:** 10.1186/s12969-016-0132-2

**Published:** 2017-01-31

**Authors:** Kristiina Aalto, Pekka Lahdenne, Kaija-Leena Kolho

**Affiliations:** Helsinki University Central Hospital, Children’s Hospital, University of Helsinki, Helsinki, 00029 HUCH Finland

**Keywords:** Abdominal pain, Biological markers, Child, Diagnosis, Gut, IBD

## Abstract

**Background:**

Patients with juvenile idiopathic arthritis (JIA) on non-steroidal anti- inflammatory drugs (NSAIDs) may experience abdominal pain. In adults, NSAID use has been linked to an increase in fecal calprotectin (FC) levels, a surrogate marker for gut inflammation. In JIA, data on gut inflammation related to drug use is scarce.

**Methods:**

JIA patients followed up at the outpatient pediatric rheumatology clinic in Children’s Hospital, Helsinki University Hospital, Helsinki, Finland were routinely assessed for FC if they complained about abdominal pain, had an elevated erythrocyte sedimentation rate (ESR) or used NSAIDs on a daily basis. The FC levels were related to the presence of abdominal pain, to ESR, and to the presence of HLA-B27.

**Results:**

Of the total group of 90 patients (median age 9.1 years; 45 JIA patients with disease modifying anti-rheumatic drugs (DMARDs), 25 without DMARD medication, and 20 arthralgia patients as controls), approximately 50% used NSAIDs, of whom 40% complained about abdominal pain. In patients with abdominal pain, one-third had elevated FC values (>100 μg/g). The FC values, for the most part, declined along with the discontinuation or reduction of NSAIDs and after intensifying the DMARD medication, where after the pain disappeared. In patients with an elevated ESR, the FC values and ESR normalized in parallel. The presence of HLA-B27 was not associated with FC levels.

**Conclusion:**

In patients with JIA and abdominal pain, it may be useful to determine the FC when evaluating the need for further gastrointestinal examinations.

## Background

In patients with juvenile idiopathic arthritis (JIA), a chronic joint inflammation of childhood, non-steroidal anti-inflammatory drugs (NSAIDs) are frequently needed. When the disease activity is low, NSAIDs can be used as a monotherapy for up to two months [1]. However, it has been reported that JIA patients may experience abdominal pain related to NSAID use [2]. NSAIDs may cause intestinal damage in adults [3], but there are less data about children [4, 5]. The differential diagnosis of abdominal symptoms, however, is challenging. On the other hand, a considerable proportion of patients with inflammatory bowel disease (IBD) may have joint pains [6, 7].

To assess the presence of inflammation in the gut in detail, endoscopy is needed. However, especially in children this procedure is challenging; it is invasive and has to be performed under anesthesia. Thus, during recent years, the use of surrogate markers of gut inflammation, such as fecal calprotectin (FC), has emerged as a valuable screening tool [8–12]. In active IBD, the correlation between the endoscopy results and FC levels is good [9–11, 13]. Several reports have shown that NSAID use in adults may result in an increase in FC levels, reflecting intestinal inflammation, and furthermore, the FC levels decline when the drug is discontinued, suggesting healing of the gut mucosa [14].

Calprotectin, a major cytosolic protein released during the activation of neutrophils, is a relatively new marker for inflammatory processes in the endothelium [15]. An increase in the serum calprotectin level is seen in several inflammatory conditions, including JIA [16]. Calprotectin is stable in feces, and fecal concentrations reflect not only colonic inflammation, but the presence of small bowel involvement as well [9, 10]. FC levels are high among patients with active IBD, providing a diagnostic tool for differentiating between recurrent abdominal pain and chronic inflammatory disease in the intestine [11, 12, 17] and reducing the need of referrals for further investigations [18]. The significance of minor elevations in FC levels is poorly understood, but dysfunctional pain is not related to FC values [12]. Constipation may be associated with a slight elevation in FC values [19].

In patients with JIA, data on the performance of FC levels in the assessment of intestinal inflammation are limited. At our hospital, we have routinely used FC measurements for several years to screen for the presence of gut inflammation in paediatric patients with non-specific abdominal symptoms, e.g. abdominal pain. The aim of this study was to evaluate the usefulness of FC measurements in patients with JIA associated with NSAID use and abdominal pain.

## Methods

A group of 90 patients followed up at the outpatient clinic of pediatric rheumatology in Children’s Hospital, Helsinki University Hospital, Helsinki, Finland, a tertiary care hospital, were routinely assessed for FC when they complained about abdominal pain, had an elevated erythrocyte sedimentation rate (ESR) or used NSAIDs on a daily basis. Of the 90 patients, 50 had JIA for at least 6 months, 20 patients had newly diagnosed JIA (Table 1). As controls we had 20 patients with arthralgia who were referred to our hospital for evaluation of JIA (Table 1). No patients with systemic-onset JIA were included and there were no patients with psoriasis or undifferentiated arthritis.

**Table 1:** Clinical characteristics of the patients; 70 having the JIA diagnosis and 20 arthralgia

All patients *n* = 90	Oligo *n* = 33	Poly *n* = 29	JSpA *n* = 8	All JIA *n* = 70	Arthralgia *n* = 20
Age	10.2 ± 3.8	8.1 ± 3.5	11.8 ± 3.0	9.6 ± 3.9	8.7 ± 4.9
Female	25 (57%)	19 (43%)	0	44 (63%)	13 (65%)
ANA-Ab +	4 (12%)	6 (21%)	0	9 (13%)	1 (5%)
Anaemia	10 (30%)	9 (31%)	3 (38%)	22 (31%)	1 (5%)
HLA-B27 +	9 (27%)	6 (21%)	7 (88%)	22 (31%)	5 (25%)
Active disease	8 (24%)	9 (31%)	0	17 (24%)	0
NSAID +	15 (45%)	14 (48%)	5 (63%)	34 (49%)	12 (60%)
DMARD +	20 (61%)	20 (69%)	5 (63%)	45 (64%)	0
Biologicals +	3 (10%)	2 (7%)	0	5 (7%)	0

*JIA* juvenile idiopathic arthritis

*Oligo* juvenile oligoarthritis, *Poly* juvenile polyarthritis (RF-negative), *JSpA* juvenile spondyloarthritis (including enthesitis related arthritis)

*ANA-Ab* antinuclear antibodies

*DMARD* disease-modifying anti-rheumatic drug

mainly methotrexate (MTX), other DMARDS: leflunomide, sulphasalazine (SSZ), hydroxychloroquine, cyclosporine A (CyA)

- Ten patients had ≥1 DMARD (usually MTX + SSZ), one patient had CyA with SSZ

*NSAID* non-steroidal anti-inflammatory drug

JIA diagnosis was based on the criteria of the International League of Associations for Rheumatology (ILAR) [20]. At the time of JIA diagnosis, the presence of HLA-B27, serum antinuclear antibodies (S-ANA) and rheumatoid factor were routinely determined. The disease was considered active (Table 1) if there were active joints in the clinical examination and the doctor’s global assessment of the disease using a visual analogue scale (VAS) was > 1 (range 0–10) or if the ESR was higher than 20 mm/h, which is comparable to a JIA disease activity score of JADAS10 > 2 [21].

### Fecal calprotectin (FC)

The FC levels were analyzed using a quantitative, enzyme-linked immunoassay (PhiCal Test, Calpro AS, Oslo, Norway) in routine analyses of the central laboratory services of the Helsinki University Hospital. The fecal samples were stored at –20°C until analyzed. FC values <100 μg/g in the stools were considered normal and values <50 μg/g very low, whereas values >1000 mg/g were found to be exceedingly high [22–24]. Measurements of FC levels were made on clinical grounds, i.e. when the patient complained of abdominal pain or had an elevated ESR or was using NSAIDs on a daily basis. Elevated FC levels were controlled at regular follow-up visits until normal.

Endoscopies were performed when the FC value was > 200-300 μg/g. When the FC value was only mildly or moderately elevated with no major symptoms, NSAIDs were discontinued.

### Statistical analyses

Data are presented as means (± standard deviation), or as ranges and medians, as appropriate. Correlations between the parameters were calculated using either a Spearman rank correlation, the Kruskall-Wallis test, or logistic regression (with a threshold of elevated FC value <100 μg/g or <50 μg/g), when appropriate and statistical comparisons between groups were performed using the Student’s *t*-test (normal distribution) or the Mann-Whitney *U* test. Statistical analyses were performed using Microsoft® software Excel 2010 or with IBM® SPSS® Statistics 19 software. A *p*-value <0.05 was considered significant.

## Results

### FC values in the patients

Of the 70 patients with JIA, 45 (64%) were on DMARD medication (Table 1). In these 45 patients, FC values ranged from 1 to 1617 μg/g (Table 2): ten (22%) had elevated FC values (>100 μg/g), six had FC values between 50 and 100 μg/g, and 29 had FC values less than 50 μg/g. JIA patients with DMARDs had significantly more often a FC value > 50 μg/g than patients without DMARDs (*p = 0.029*). Of the 20 arthralgia patients (Table 2) one (5%) with abdominal pain had a mildly elevated FC value of 113 μg/g (Fig. 2) which returned to normal two months later. Twelve patients with arthralgia used NSAIDs on a daily basis.

**Table 2:** Diagnoses of the patients and proportion of patients with abdominal pain, NSAID usage, and their ESR and FC values

All patients (*n* = 90)	Abdominal pain *n* (%)	NSAID usage *n* (%)	ESR mm/h range (median) elevated (*n*)	FC μg/g range (median) elevated (*n*)
JIA patients				
Oligoarthritis (33)	13 (39%)	15 (45%)	1–80 (12) *n* = 15	1–368 (17) *n* = 5
Polyarthritis (29)	11 (38%)	14 (48%)	1–94 (16) *n* = 15	2–1617 (34) *n* = 7
JSpA* (8)	1 (13%)	5 (63%)	10–35 (19) *n* = 4	11–184 (18) *n* = 1
All JIA (70)	27 (39%)	34 (70%)	1–94 (15) *n* = 34 (49%)	1–1617 (25) *n* = 13 (19%)
Arthralgia (20)	12 (60%)	12 (60%)	7–50 (10) *n* = 7 (35%)	0–113 (32) *n* = 1 (5%)

*JIA* juvenile idiopathic arthritis

*JSpA juvenile spondylarthritis

*NSAID* non-steroidal anti-inflammatory drug

*ESR* erythrocyte sedimentation rate

*FC* fecal calprotectin, cut-off of 100 μg/g for elevated values [17–19]

**Fig. 1:**
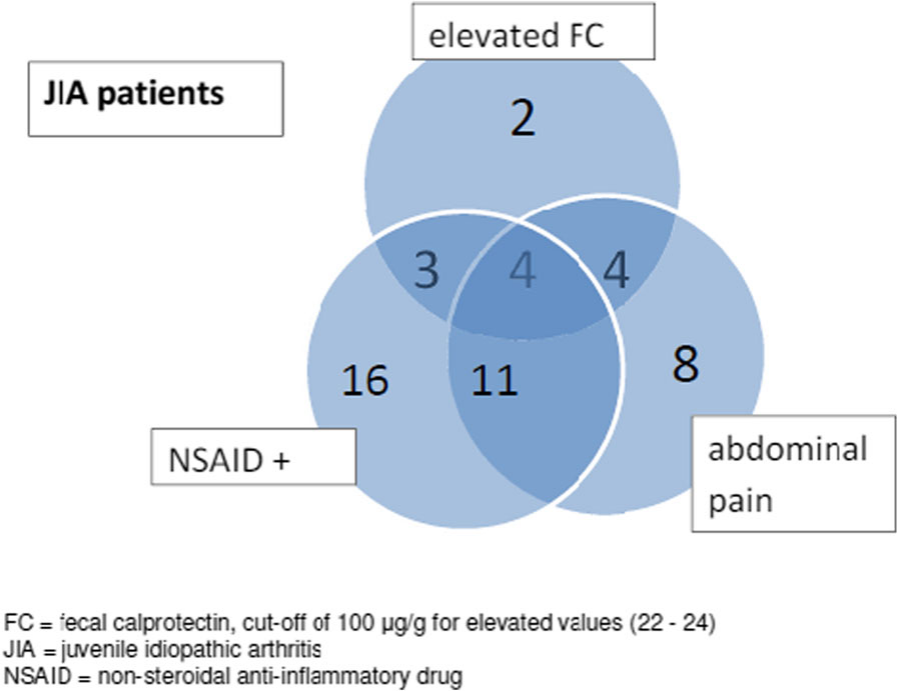
JIA patients with elevated FC-values and/or regular NSAID use and/or abdominal pain. Of the 70 patients with JIA 48 had elevated FC value and/or NSAID use and/or abdominal pain. The numbers in overlapping areas show the combinations e.g. JIA patients having both abdominal pain and elevated FC values (*n* = 8), and four of them used NSAIDs as regular bases. See the text for other details

Of the ten JIA patients on DMARDs and with elevated FC values, seven presented with abdominal pains (Tables 2 and 3, Fig. 1) and five used NSAIDs on a regular basis. Three patients underwent endoscopies. One of them had an exceedingly high FC value (1617 μg/g). This particular patient reported abdominal pain, and after medication for endoscopy-confirmed esophagitis with a proton pump inhibitor (PPI) and dietary adjustment, her pains disappeared and the FC value declined after three months. Two other patients underwent endoscopies because of abdominal pain and elevated FC values (both with values > 200 μg/g). In these two, biopsy findings revealed mild gastritis and irritation in the bowel but no pathological changes in the capsule endoscopy of the small intestine. Naproxen was discontinued, and abdominal pain subsequently disappeared in both patients. FC-values normalized in two to three months.

**Table 3:** FC-values of the JIA patients in relation to their abdominal pain, NSAID usage, and ESR values

All JIA patients (*n* = 70)	Abdominal pain	NSAID usage	elevated ESR >20 mm/h
FC 0–50 μg/g (*n* = 51)	38%	43%	41%
FC 50–100 μg/g (*n* = 6)	50%	83%	83%
FC 100–200 μg/g (*n* = 10)	50%	60%	70%
FC > 200 μg/g (*n* = 3)	100%	67%	67%

*JIA* juvenile idiopathic arthritis

*NSAID* non-steroidal anti-inflammatory drug

*ESR* erythrocyte sedimentation rate

*FC* fecal calprotectin

The remaining seven JIA patients using DMARDS and with FC values >100 μg/g (all with values less than 200 μg/g) did not undergo endoscopies (Tables 2 and 3). Of these patients, two suffered from constipation. After successful treatment of constipation, their FC values fell within normal ranges. The five other patients were advised to take NSAIDs only for short periods when they used NSAIDs on daily bases. Their FC values returned to normal after restricting NSAID use in all except one. In this particular patient, the FC values fluctuated between 89 and 181 μg/g during the follow-up even when he was not on NSAIDs. He also had a diagnosis of IgA deficiency and autoimmune (AI) hepatitis.

FC values were between 50 and 100 μg/g in six JIA patients with DMARDs, of whom three suffered from abdominal pain. These three had an active joint disease and used NSAIDs regularly. Several months later, one of them had an even higher FC value, 258 μg/g, but after reducing the amount of NSAIDs he was taking and increasing the dosage of DMARDs his abdominal pain disappeared, and the FC value declined to normal after two months. Similarly, when the joint disease of the other two patients was better controlled, the patients’ need for NSAIDs decreased, and the FC values fell below 50 μg/g also after three to four months.

Twenty-five patients with JIA were not on DMARDS, and the majority (*n* = 20) of them had a disease duration of less than 6 months. The FC values ranged from 2 to 189 μg/g (Fig. 1, Table 3). Of the 25 patients three (12%) had elevated values (>100 μg/g), and the rest 88% had very low FC values (<50 μg/g). The three patients with elevated FC values used NSAIDs daily (Fig. 1). They also presented with some functional gastrointestinal symptoms, but only one reported abdominal pain.

### FC values related to disease characteristics, abdominal pain, and NSAID usage

Patients with JIA had significantly more often elevated FC value (*p* = 0.017) than arthralgia patients. The presence of ANA antibodies or HLA-B27 was not associated with the elevation of FC, nor was the subtype of JIA.

**Fig. 2:**
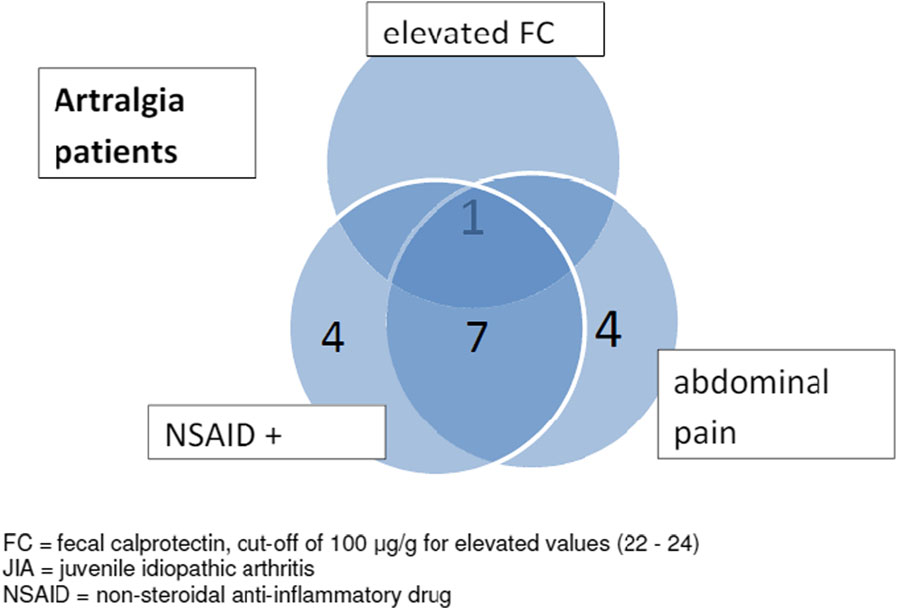
Arthralgia patients with elevated FC-values and/or regular NSAID use and/or abdominal pain. Of the 20 patients with arthralgia 12 had abdominal pain of which eight used NSAIDs (overlapping areas). See text for details

In total, 27 (39%) of the 70 patients with JIA and 12 (60%) of the 20 with arthralgia experienced abdominal pain (Figs. 1 and 2, Table 2). There was a linked association between abdominal pain, elevated ESR values and elevated FC values (overall *p = 0.001*). Of the 14 patients (13 with JIA and one with arthralgia) with elevated FC values (>100 μg/g, Tables 2 and 3), nine had abdominal pains and six of them had elevated ESR values. In patients with an elevated ESR, anti-rheumatic treatment was intensified which led to a decline of both the FC and ESR (data not shown).

Of the 29 JIA patients on DMARDs who had FC values of less than 50 μg/g, 11(38%) reported some abdominal pain. All except one were girls, aged 5 to 15 years, and they all had a tendency to experience other non-specific pains as well, such as limb pain and headaches.

About half of the JIA patients (*n* = 34) used NSAIDs regularly, and 15 (44%) of them reported abdominal pains. Among the patients taking NSAIDs daily and experiencing abdominal pain, one third had elevated FC values >100μg/g (Fig. 1, Table 2). In the whole group of 90 patients, the use of NSAIDs was associated with elevated FC levels (*p = 0.03*). Even though there was no clear correlation between NSAID use and abdominal pain, in many cases, abdominal pains lessened after lowering the NSAID dosage. At the same time the DMARD medication of the JIA patients was intensified.

If the threshold of elevated FC value was lowered to 50 μg/g and a multivariate analysis made, the correlation of NSAID use came more evident. Logistic regression analysis showed that NSAID (OR = 3.96) and DMARD (OR = 3.78) use together with abdominal pain (OR = 3.96) were significantly (overall *p = 0.046*) correlated to the FC elevation. Of these three parameters abdominal pain associated most strongly to the elevated FC-value.

In the JIA group, 24% had an active joint disease. Disease activity was not associated with abdominal pain or the elevated FC value (data not shown). None of the patients had celiac disease or food allergies; each child tolerated milk, except for two, one was lactose intolerant and one with a cow’s milk allergy and Down’s syndrome.

## Discussion

JIA patients who were on DMARDs had significantly higher FC values than the other JIA patients, possibly suggesting intestinal inflammation. The difference, however, seemed to be associated with NSAID use, too. The higher FC values in those patients who used NSAIDs regularly returned to normal after tapering the NSAID dosage. Concurrently, in most JIA patients, DMARD medication was also intensified. Thus, it can be speculated that JIA patients may have some low activity also in the intestinal mucosa when their joint disease is not adequately controlled.

A recent comprehensive study in 44 adult spondyloarthritis (SpA) patients showed that FC can be elevated even without obvious GI symptoms [25]. There are no such studies of children. As reported in adults, the use of NSAIDs may induce a modest increase in the FC values, but rarely above 300 μg/g [26], which is in line with our findings. In a recent study [27] of adult rheumatoid arthritis (RA) and osteoarthritis (OA) patients using NSAIDs small intestinal lesions were identified significantly more often in RA (56.8%) patients compared to OA (31.9%) suggesting a possible influence of the underlying disease. They did not find any laboratory marker (hemoglobin, ESR, or fecal occult blood test) to be used as a diagnostic factor but they did not test FC. In our study the arthralgia patients had clearly lower FC values suggesting also that the underlying disease might have an explanatory role and not only the NSAID usage.

In our study most patients with higher FC values had abdominal pain, but the presence of pain varied among patients with lower FC values. The FC levels in patients without GI problems were comparable to those observed in healthy children [28]. However, our upper normal limit (<100 μg/g) is somewhat higher when compared to the manufacturer’s instructions (50 μg/g) and several other studies with a different immunoassay [18, 28]. Since it has been suggested that pediatric patients having FC values greater than 50μg/g should be monitored for a possible flare-up of intestinal inflammation [28], we also stratified the patient groups according to a lower cut-off level for FC values (>50μg/g). With this lower cut-off level, the JIA patients on NSAIDs showed significantly more elevated values than other patients. These patients with elevated FC values had significantly more often also abdominal pain and DMARD medication.

When the joint disease was active patients took NSAIDs more regularly, but as such, the presence of active joint inflammation was not associated with an increase in FC values. When the joint disease was appropriately controlled and NSAIDs less often needed, the patients did not report abdominal pain. Their FC values were not elevated anymore in the control samples taken approximately two months afterwards. The fact that only five JIA patients in our study group were taking biological DMARDs indirectly shows that patients using biologics have such a low disease activity that they did not fulfill the criteria of this study (elevated ESR, NSAID usage or abdominal pain). Also, in Finland, we apply an aggressive step-down treating model [29] and therefore, most of the JIA patients used DMARDS. There is some evidence that biologics, especially etanercept, could provoke the development of IBD [30]. While three (out of five) of our patients on biologics used etanercept and had low FC values, the number of patients was too low to draw any conclusions. Recent reports suggest that patients with SpA using etanercept confer a risk of IBD [31], but we had no such patients.

All subgroups of JIA patients presented with comparable FC values, though the polyarthritis patients tended to have higher values. These higher values in polyarthritis patients were again mainly explained by NSAID use in the active joint disease. Among HLA-B27 positive JIA patients, there is a risk for evolution of IBD [32, 33], but we did not find such patients with a suspicion of IBD. Here, the proportion of HLA-B27 positive patients was quite high, though in line with earlier studies from Finland and the Nordic countries [34, 35]. In contrast, a recent study reported higher FC levels among patients with enthesitis-related arthritis (ERA, HLA-B27 positive juvenile SpA) than among other JIA patients [36]. This difference was not explained by NSAID use. However, the study reported that only one out of nine ERA patients with abdominal pain had an elevated FC value (>300 μg/g). In a recent pilot study with adult patients, elevated FC levels (cut-off value >50 μg/g) were found in 41% (of 39) patients with ankylosing spondylitis [37]. In a more recent study, the optimal cut-off value for FC detecting bowel inflammation in patients with ankylosing spondylitis was 85 μg/g [20]. In that study, the FC values were higher in patients with NSAID use, but the association with bowel inflammation remained after adjustment for the drug use. These results may indicate that ERA/SpA patients should be under careful observation for possible gut damage. The present results in pediatric patients do not support this suggestion because only one HLA-B27 positive patient had FC value greater than 100 μg/g. On the other hand, the total number of HLA-B27 positive JIA patients was limited, and more studies on this matter need to be conducted in children.

When the FC value is exceedingly high, endoscopies are recommended, but with lower values (150–300 μg/g) and minor abdominal symptoms modifying the medication and/or diet may be the primary options. One of our JIA patients had a very high FC value, which declined below the cut-off point after introducing medication for esophagitis confirmed in endoscopy and adjusting the dairy consumption. Endoscopies are invasive procedures requiring anesthesia in pediatric patients with indications to be discussed with pediatric gastroenterologists.

Taken together, we found that those JIA patients who needed NSAIDs for longer periods have often elevated FC levels. Notably, this might be a sign of suboptimal disease control. In adults e.g. chronic gastritis did not cause an increase in FC values [38]. Whether an uncontrolled joint disease could increase the risk of gut involvement remains to be seen in future studies. In the present study, one fifth of the JIA patients had an elevated FC value; none of them had signs of IBD, and only a few of the patients fulfilled indications for an endoscopy.

The fecal test was easy to carry out, and the patients readily agreed to bring a sample with them. Some patients had abdominal pains but a low FC value; those patients also had other non-specific aches and joint pains that could not be attributed to arthritis, representing the possibility of a dysfunctional pain disorder [39]. For patients with low FC levels, patients taking NSAIDs and patients experiencing minor pains, invasive and unnecessary endoscopies could be avoided [40]. Abdominal pains are common, and while another study found that 14–16% of 8-year-old Finnish children experience abdominal pain [41], in our study approximately 44% of the patients reported such pain. Notably, FC levels are low among patients with functional abdominal pain [42].

## Conclusions

To conclude, we recommend determining the FC values of JIA patients who complain of abdominal pain or who regularly take NSAIDs for several weeks. If the FC value is high and remains high when cutting down exposure to NSAIDs, bowel examinations (endoscopies) should be considered. As the FC test is reliable in ruling out intestinal inflammation and can easily be repeated, it should be used more often because it is a simple non-invasive procedure for the patient.

## Key messages

1. Fecal calprotectin, a surrogate marker of gut inflammation, should be measured in JIA patients complaining abdominal pain or using NSAIDs regularly.
2. When the fecal calprotectin value is consistently elevated, consider the need of endoscopies.
3. A fecal calprotectin test is a useful tool in screening for the need of gastrointestinal investigations.

## Abbreviations

AI: Autoimmune
ANA: Antinuclear antibodies
CyA: Cyclosporine A
DMARDs: Disease modifying anti-rheumatic drugs
ERA: Enthesitis-related arthritis
ESR: Erythrocyte sedimentation rate
FC: Faecal calprotectin
GI: Gastrointestinal
IBD: Inflammatory bowel disease
ILAR: International League of Associations for Rheumatology
JADAS: JIA disease activity score
JIA: Juvenile idiopathic arthritis
JSpA: Juvenile spondylarthritis
MTX: Methotrexate
NSAIDs: Non-steroidal anti-inflammatory drugs
oJIA: Oligoarthritis
pJIA: Polyarthritis
PPI: Proton pump inhibitor
S-ANA: Serum antinuclear antibodies
SpA: Spondyloarthritis
SSZ: Sulphasalazine
VAS: Visual analogue scale
